# Pancreatic Pseudocyst With Thoracic Extension: A Clinicoradiological Case Report

**DOI:** 10.1002/ccr3.71745

**Published:** 2025-12-29

**Authors:** Sachchu Thapa, Bijay Kunwar, Anup Ghimire, Manthan Sanjay Shinde, Ashok Kushwaha, Arjun Gaire, Jagdish Kunwar, Bikash Bikram Adhikari

**Affiliations:** ^1^ Department of Radiodiagnosis National Academy of Medical Sciences, Bir Hospital Kathmandu Nepal; ^2^ Tribhuvan University Institute of Medicine, Maharajgunj Medical Campus Kathmandu Nepal; ^3^ Department of Pediatrics, National Academy of Medical Sciences, Kanti Children's Hospital Kathmandu Nepal

**Keywords:** mediastinal pseudocyst, pancreatic pseudocyst, pancreatitis, pleural effusion

## Abstract

Pancreatic pseudocysts are encapsulated, enzyme‐rich peripancreatic fluid collections that typically develop following acute or chronic pancreatitis due to pancreatic ductal disruption. While commonly localized to the lesser sac, rare mediastinal extension may occur, presenting with nonspecific thoracic symptoms such as chest pain, dyspnea, or dysphagia. Cross‐sectional imaging (CT/MRI) is essential for diagnosis. Management is individualized, ranging from conservative medical therapy to endoscopic, percutaneous, or surgical drainage based on symptom severity, complications, and anatomical considerations. We report the case of a 26‐year‐old male with a history of alcohol use and smoking, presenting with cough and dyspnea. Clinical examination revealed tachypnea, hypoxia, and signs of right‐sided pleural effusion. Chest X‐ray revealed complete opacification of the right hemithorax. Diagnostic thoracentesis yielded amylase‐rich pleural fluid (11,545 IU/L). Serum amylase and lipase were also elevated. Contrast‐enhanced CT imaging demonstrated acute necrotizing pancreatitis with peripancreatic collections extending into the thoracic cavity via the esophageal hiatus, confirming a pancreatic pseudocyst with secondary massive right‐sided amylase‐rich pleural effusion. The patient was managed conservatively with antibiotics, octreotide, and ultrasound‐guided pigtail catheter drainage. The clinical course was favorable, with complete symptomatic resolution and no evidence of recurrence on follow‐up. This case underscores a rare but significant thoracic complication of acute pancreatitis manifesting as massive pleural effusion, mimicking thoracic pathology. Thus, accurate diagnosis using contrast‐enhanced CT and MRCP, along with tailored management from conservative therapy to invasive drainage, is crucial. Early recognition and multidisciplinary care ensure favorable outcomes, as highlighted in this case.

## Key Clinical Message

Unexplained massive pleural effusion with pleural amylase > 1000 U/L should prompt evaluation for a pancreatic source. A mediastinal pseudocyst, though rare, is an important differential diagnosis. In patients with unexplained respiratory symptoms, especially with a history of pancreatitis or alcohol use, pancreatic origin should be considered.

## Graphical abstract

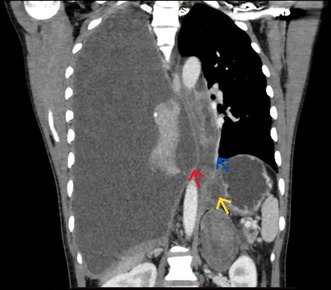

**Abbreviations**
AFB: acid‐fast bacilli
CECT: contrast‐enhanced computed tomography
CT: computed Tomography
ERCP: endoscopic retrograde cholangiopancreatography
EUS: endoscopic ultrasound
FNA: fine‐needle aspiration
MMRC: modified medical research council
MRCP: magnetic resonance cholangiopancreatography
MRI: magnetic resonance imaging
USG: ultrasonography

## 1 Background

A pancreatic pseudocyst is a localized, peripancreatic fluid collection rich in amylase and lipase, surrounded by a well‐defined fibrous wall, typically developing more than 4 weeks after an episode of acute pancreatitis [1, 2, 3]. Pancreatic pseudocysts are collections that communicate with the pancreatic duct system, either directly or through the surrounding pancreatic tissue. They typically develop due to disruption of the pancreatic ducts, which can occur from increased ductal pressure caused by strictures, stones, or protein plugs blocking the main duct [4]. Pancreatic pseudocysts, which can develop as a complication of pancreatic necrosis following acute pancreatitis, may occur in both acute and chronic pancreatitis, with reported incidences of 7%–25% and 20%–40%, respectively [4, 5]. Pancreatic pseudocysts are usually well‐circumscribed and located outside the pancreas, most commonly within the lesser sac; while they are often asymptomatic, they can occasionally extend into unusual sites such as the mediastinum, neck, or even the testes [5]. The reported incidence of mediastinal extension is very rare [5].

Mediastinal extension of a pancreatic pseudocyst presents with atypical symptoms including chest pain, dysphagia, nausea and vomiting, dyspnea, cough, hemoptysis, and palpitation [5]. Primarily found pseudocysts are mostly asymptomatic. Cross‐sectional imaging (CT and MRI) is the method of choice in identifying and diagnosing mediastinal extension of a pancreatic pseudocyst [6]. Management of pancreatic pseudocysts depends on the individual case and may range from observation and medical therapy to endoscopic or percutaneous drainage, or surgical removal when necessary.

## 2 Case Presentation

### 2.1 Case History

We present a case of a 26‐year‐old male, known smoker and alcohol consumer, with no known prior co‐morbidities, with complaints of cough and shortness of breath. Cough had been present for the past 20 days, acute onset, dry in nature, and intermittent. There wasn't associated fever, chest pain, abdominal pain, vomiting, or weight loss. He reported shortness of breath corresponding to Modified Medical Research Council (MMRC) Grade 2. There was no history of orthopnea, paroxysmal nocturnal dyspnea (PND), palpitations, chest pain, limb swelling, abdominal pain, or decreased urine output.

On examination, he was alert and oriented to time, place, and person. He had tachypnea, tachycardia, and saturation of O_2_ 84% on room air, requiring 2 L O_2_. Chest exam showed decreased right‐sided chest wall movement, tracheal deviation to the left, dull percussion over the right hemithorax, and absent breath sounds on the right. Other systemic and abdominal exams were unremarkable. Findings suggest underlying pathology affecting right lung expansion, possibly due to pleural effusion, lung collapse, or central airway obstruction.

### 2.2 Investigations

**FIGURE 1:**
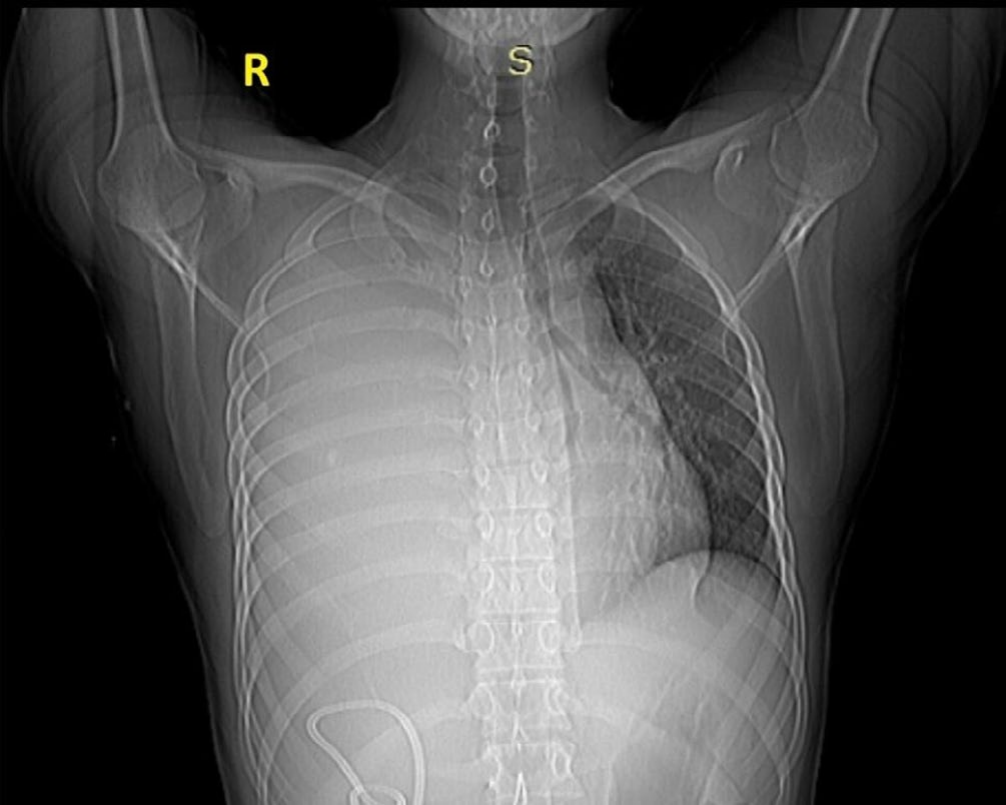
Chest X‐ray (Posteroanterior view) shows a large right pleural effusion causing leftward mediastinal shift, with a pigtail drainage catheter in situ.

All examination findings collectively point toward a significant right‐sided intrathoracic abnormality, most likely massive pleural effusion or lung collapse with mediastinal shift to the contralateral side. Serial baseline investigations including complete blood count (CBC), LFTs, and renal function tests (RFT) were performed shown in Table 1 and (Data S1). A chest X‐ray revealed complete opacification (“white‐out”) of the right hemithorax, suggestive of a large pleural effusion or massive lung collapse as shown in Figure 1. The mediastinal shift to the left was also noted, supporting the clinical suspicion of significant right‐sided intrathoracic pathology. Sputum analysis, including acid‐fast bacilli (AFB) smear, culture, and GeneXpert MTB/RIF assay, was performed to rule out tuberculosis, and the results were negative (Table 1). Fungal studies were also negative (Table 1). A diagnostic pleural fluid aspiration was performed, which revealed Amylase level: 11,545 U/L (markedly elevated) (Table 1). Serum Amylase (492 U/L) and lipase (192 U/L) were thrice above the baseline amylase‐rich pleural effusion, most likely secondary to pancreatitis (Table 1). Further evaluation of serum liver function tests (LFTs) showed no abnormalities, ruling out significant hepatobiliary involvement. Cytological analysis of pleural fluid for malignant cells was negative, ruling out malignancy as a cause of the effusion.

**TABLE 1:** Lab reports on the day of admission.

Test	Results	S. I. units	Reference range in SI unit
Hematology report			
CBC			
Total count			
WBC	7.5	× 10^9^/L	4.0–11.0
RBC	4.46	× 10^12^/L	4.7–6.0
Platelets	375	× 10^9^/L	150–400
Differential count			
Neutrophils	43	%	40–70
Lymphocytes	44	%	20–45
Monocytes	10	%	2–10
Eosinophils	3	%	1–6
Basophils	0	%	0–1
Hemoglobin	139	g/L	130–180
PCV	0.42	L/L	0.40–0.54
MCHC	330	g/L	280–350
MCH	28	pg	27–32
MCV	81	fL	80–96
Biochemistry report			
Blood urea	2.66	mmol/L	1.67–7.50
Serum creatinine	53	μmol/L	35–124
Sodium	129	mmol/L	135–145
Potassium	3.3	mmol/L	3.5–5.0
Serum bilirubin total	13.7	μmol/L	5–17
Serum bilirubin direct	8.6	μmol/L	0–6.8
SGPT/ALT	30	U/L	5–40
Alk. phosphatase	83	U/L	35–150
SGOT/AST	47	U/L	< 50
Serum total protein	61	g/L	45–80
Serum albumin	29	g/L	25–55
Lactate dehydrogenase	208	U/L	225–450
Serum amylase	492*	U/L	< 80
Serum lipase	198*	U/L	0–38
Microbiology report			
Sputum for gram stain	Gram positive and negative cocci, bacilli and others not seen		
Sputum for AFB A/B/GeneXpert	Not detected		
Pleural fluid analysis			
Culture sensitivity	No growth for organisms after 48 h, inoculation at 37°C		
Pleural fluid serum amylase	11,545* (fluid)	IU/L	< 80
Pleural fluid repeated analysis	15,535* (fluid)	IU/L	< 80
Pleural fluid protein	45	g/L	> 30 g/L
Pleural/Serum protein ratio	0.73*	Ratio	> 0.5
Pleural fluid LDH	420	U/L	
Pleural/Serum LDH ratio	2.02*	Ratio	
Pleural fluid glucose	2.8	mmol/L	< 3.3 mmol/L
Pleural fluid pH	7.25	pH units	< 7.3
Pleural fluid total cell count	3200*	cells/μL	> 1000

* abnormal value.

**FIGURE 2:**
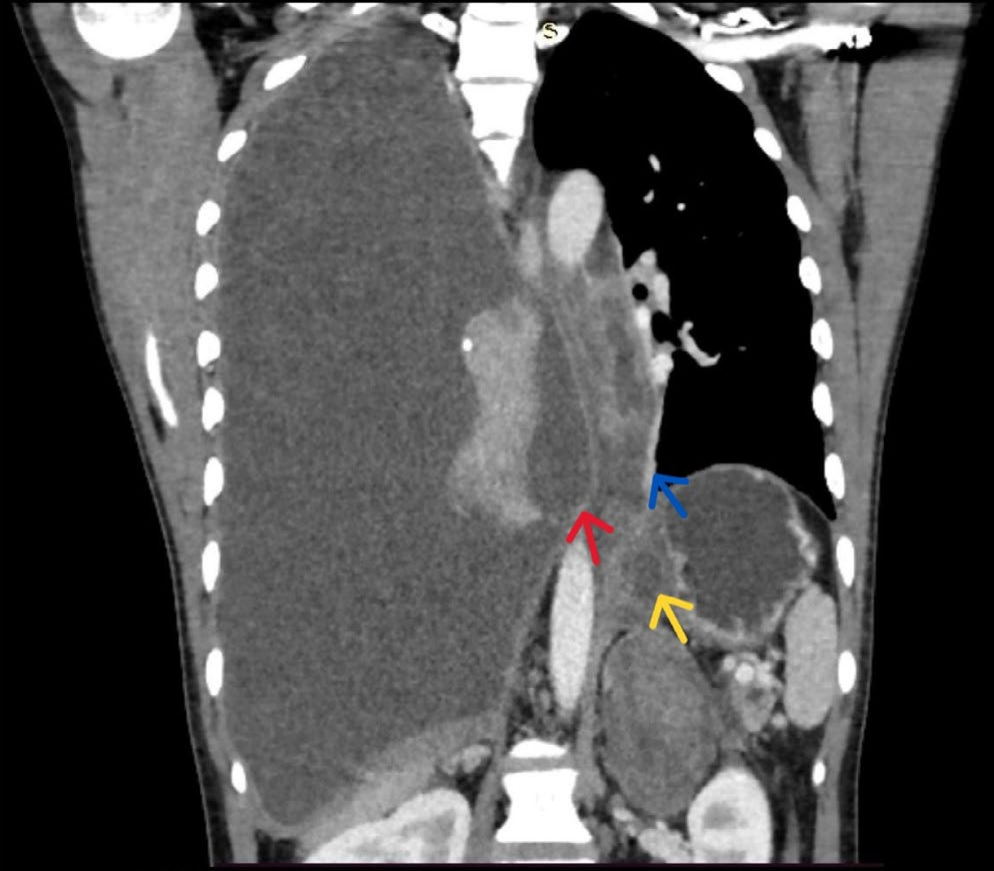
CECT sagittal view of the chest and abdomen CECT sagittal view shows an enlarged, heterogeneously enhancing pancreas with a peripancreatic collection extending into the posterior mediastinum and compressing the esophagus with massive right pleural fluid noted.

A plain and contrast‐enhanced computed tomography (CECT) of the chest, abdomen, and pelvis was performed, which revealed the following findings: *Pancreas*: Heterogeneous pancreatic parenchyma enhancement with areas of non‐enhancement consistent with acute necrotizing pancreatitis. Necrotic areas involving both pancreatic parenchyma and peripancreatic tissues. *Peripancreatic Region/Fluid Collections*: Multiple peripancreatic fluid collections present. One collection extending through the widened esophageal hiatus into the thoracic cavity, consistent with intrathoracic extension of a pancreatic pseudocyst. Estimated volume of the necrotic pseudocyst, approximately 200 mL. Associated peripancreatic lymphadenopathy noted (Figure 2). *Thorax*: Gross right‐sided pleural effusion noted, likely secondary to pancreatic pseudocyst extension. Overall Impression based on Revised Atlanta Classification [7]: (1) Acute peripancreatic fluid collection evolving into a pancreatic pseudocyst with thoracic extension through the esophageal hiatus. (2) Secondary right‐sided amylase‐rich pleural effusion due to pseudocyst extension. (3) Acute necrotizing pancreatitis (pancreatic and peripancreatic necrosis present).

Based on the above findings, a diagnosis of pancreatic pseudocyst with thoracic extension causing right‐sided pleural effusion was established. Severity assessment using imaging demonstrated a pseudocyst arising from peripancreatic collections and extending through the esophageal hiatus into the thorax, resulting in a right‐sided amylase‐rich pleural effusion, confirmed by pleural fluid analysis. The patient has moderate acute necrotizing pancreatitis (Modified CTSI 6/10) with Balthazar grade E and < 30% pancreatic necrosis. Although the BISAP score of 1 suggests low systemic risk, the presence of a pseudocyst with thoracic extension constitutes a significant local pancreatic complication, necessitating close clinical monitoring and multidisciplinary management.

## 3 Management and Counseling

The patient was managed conservatively with IV ceftriaxone for 7 days for prophylaxis and SIRS control in necrotizing pancreatitis. Octreotide (100 μg SC three times daily for 10 days) was given to reduce pancreatic secretion and aid resolution of the pseudocyst and amylase‐rich effusion. USG‐guided placement of a 12 Fr pigtail catheter relieved the massive right pleural effusion; about 1300 mL was drained over 5 days with daily monitoring. Respiratory symptoms improved steadily, and serial imaging showed complete resolution by day 14. The patient remained stable without complications and was discharged after 16 days.

On discharge, he was advised to continue medical therapy per gastroenterology guidance, including pancreatic enzyme supplementation, strict abstinence from alcohol and smoking, and adherence to a low‐fat, pancreatic‐friendly diet. He was counseled on recognizing early warning symptoms. Follow‐up in the Gastroenterology OPD at 3, 6, and 12 months showed normal clinical exams, with the patient remaining asymptomatic and compliant with lifestyle and medical recommendations.

## 4 Discussion

Pancreatic pseudocysts are known complications of both acute and chronic pancreatitis. The reported prevalence of pseudocysts in acute pancreatitis ranges from 6% to 18.5% and is higher in chronic pancreatitis, ranging from 20% to 40% [8, 9, 10]. Among the etiologies, alcoholic chronic pancreatitis remains the most common cause, accounting for approximately 70%–78% of pseudocyst cases [11]. Idiopathic chronic pancreatitis is the second most common cause, responsible for 6%–16% of cases, followed by biliary pancreatitis, which accounts for 6%–8% [11, 12]. Despite these associations, the overall incidence of pancreatic pseudocyst formation remains relatively low, estimated at 1.6%–4.5%, or approximately 0.5–1 case per 100,000 adults per year [12]. Our case was also associated with alcohol use; on retrospective history, the patient reported occasional episodes of abdominal pain, particularly following episodes of binge drinking.

In the context of acute pancreatitis, the formation of pancreatic pseudocysts is primarily attributed to disruption of the main pancreatic duct or its side branches, particularly allowing pancreatic enzymes and secretions to leak into surrounding tissues and form a walled‐off fluid collection that subsequently matures into a pseudocyst [12]. In contrast, the exact mechanism of pseudocyst formation in chronic pancreatitis remains less clearly defined [12]. It is hypothesized that, in addition to acute exacerbations of fluid leakage, obstruction of the pancreatic duct due to protein plugs or pancreatic calculi may contribute to pseudocyst formation in chronic pancreatitis [12, 13]. Interestingly, approximately two‐thirds of pseudocysts demonstrate a persistent communication with the main pancreatic duct, which may perpetuate the cyst's existence. In the remaining cases, the connection appears to be sealed off, likely due to fibrotic changes or an inflammatory reaction [13, 14]. In terms of etiology, most pancreatic pseudocysts in adults are secondary to alcohol‐induced pancreatitis, whereas in pediatric populations, abdominal trauma remains the leading cause [14].

Pancreatic pseudocysts are typically confined to the lesser sac, but mediastinal extension is a rare and unusual complication, reported in patients across a wide age range, from 7 months to 73 years [14]. Mediastinal involvement most commonly occurs when peripancreatic fluid tracks through anatomical diaphragmatic openings, particularly the esophageal or aortic hiatus, into the posterior mediastinum. Additionally, peripancreatic fluid may find alternative pathways into the middle or anterior mediastinum, including the inferior vena cava (IVC) hiatus, through a Morgagni hernia or by direct transdiaphragmatic extension due to tissue breakdown from inflammation and enzymatic digestion [7, 13, 14, 15]. In cases where rupture of the posterior pancreatic duct occurs, pancreatic fluid generally accumulates in the retroperitoneal space. However, in rare scenarios, this fluid can migrate into the posterior mediastinum via the esophageal or aortic hiatus, resulting in mediastinal pseudocyst formation [15]. Similarly, if the fluid transgresses the IVC hiatus or the foramen of Morgagni, it can present as a middle mediastinal or anterior mediastinal pseudocyst, respectively [15]. In the reported case, the collection communicated to the thoracic cavity through a widened esophageal hiatus.

Pancreatic pseudocysts can often mimic other extra‐pancreatic conditions, making diagnosis challenging. These include peptic ulcer disease, gastric and ovarian malignancies, acute myocardial infarction (MI), intestinal obstruction, pneumonia, intra‐pancreatic neoplasms, and vascular pseudoaneurysms [13]. The typical presentation of a conventional pancreatic pseudocyst includes symptoms like abdominal pain, early satiety, nausea, vomiting, jaundice, and occasionally bleeding [7]. In contrast, mediastinal pseudocysts are rare and often lack typical pancreatic symptoms, instead presenting predominantly with cardiopulmonary and upper gastrointestinal signs such as dysphagia, palpitations, chest pain, pleural effusion with dyspnea, cough, and hemoptysis; in severe cases, they may cause life‐threatening complications including hemothorax, cardiac tamponade, esophagobronchial fistula, sepsis, and acute airway obstruction, which can be fatal if not promptly treated [16, 17, 18]. Given the atypical presentation and variety of possible symptoms, a high index of suspicion is essential, particularly in cases like ours, where the symptoms do not follow the classical pattern of pancreatitis or pseudocyst formation.

Computed Tomography (CT) has a superior sensitivity of 90%–100% for detecting pancreatic pseudocysts, making it the gold standard imaging modality for initial diagnosis [19, 20]. CT, especially contrast‐enhanced CT (CECT), is highly effective in diagnosing pseudocysts and usually does not require any additional investigations to confirm the diagnosis [13]. It remains the primary diagnostic tool for assessing the presence, size, and location of pseudocysts. In contrast, Ultrasonography (USG) has a sensitivity range of 75%–90% [4, 19], making it less reliable for initial diagnosis but useful for follow‐up of asymptomatic pseudocysts or when the diagnosis is uncertain. Magnetic Resonance Imaging (MRI) and Magnetic Resonance Cholangiopancreatography (MRCP) offer excellent diagnostic accuracy, especially for visualizing pancreatic ductal anatomy and detecting ductal abnormalities; however, they are not routinely used for the initial diagnosis of pancreatic pseudocysts but may be employed selectively to further characterize the cyst, useful in demonstrating direct communication between the main pancreatic duct and the mediastinal pseudocyst, which may help confirm the origin of the collection [21, 22, 23]. MRI/MRCP imaging is also valuable in excluding other potential causes of a posterior mediastinal mass, such as esophageal neoplasm, bronchogenic cysts, aortic aneurysms, or hiatal hernias, helping rule out differential diagnoses. In cases where a differential diagnosis is still unclear [10], transesophageal fine‐needle aspiration (FNA) can be performed to obtain a sample for cytology, although medical history and CT imaging findings are usually sufficient to establish a diagnosis [24]. Although endoscopic options such as ERCP with ductal stenting or EUS‐guided drainage are effective for pancreatic fluid collections, these were not pursued as the patient remained clinically stable with progressive radiological resolution under conservative management. There was no evidence of persistent ductal disruption warranting intervention, and the high procedural cost made endoscopic therapy unaffordable in the mentioned case. Following the recommended step‐up approach [25], conservative management with close follow‐up was deemed appropriate and agreed upon with the patient and family.

Massive right pleural effusion has many causes, including malignancy, tuberculosis, hepatic hydrothorax, pulmonary embolism, and chylothorax. In this patient, malignancy was excluded by negative cytology and CT findings; tuberculosis by negative AFB smear, culture, GeneXpert, and absent systemic or parenchymal signs; hepatic hydrothorax by normal liver tests and imaging without cirrhosis or ascites; and pulmonary embolism by normal CT and absence of thromboembolic signs. The serosanguinous, amylase‐rich fluid with normal triglycerides also ruled out chylothorax. Based on CECT findings, pleural fluid analysis, and clinical correlation, a pancreatic pseudocyst with thoracic extension causing the right pleural effusion was diagnosed in the mentioned case.

The management of pancreatic pseudocysts with mediastinal extension requires a multidisciplinary approach, tailored to the patient's clinical presentation, imaging findings (size, location, and complications), and available expertise [3]. While most peripancreatic pseudocysts tend to resolve spontaneously, the spontaneous resolution of pseudocysts with mediastinal extension is exceptionally rare [7]. Only a few isolated cases of mediastinal extension's spontaneous resolution have been reported in the literature [26]; thus, most cases necessitate some form of intervention. For clinically stable or minimally symptomatic patients, conservative management is often the initial approach, which includes strict dietary modification, preferably a low‐fat diet, pancreatic enzyme supplementation to reduce pancreatic exocrine stimulation, complete abstinence from alcohol, especially in alcohol‐related pancreatitis cases [5]. Pharmacological therapies have also been reported, including bromhexine hydrochloride and somatostatin analogues such as octreotide, which reduce pancreatic secretions and facilitate the healing of ductal disruptions [27, 28]. Octreotide has been administered successfully at a dose of 0.5 mg subcutaneously three times daily for up to one month, with favorable outcomes reported [29].

In patients presenting with persistent symptoms such as epigastric pain, vomiting, jaundice, or signs of obstruction or compression, interventional drainage procedures are warranted [30]. Options include CT‐guided percutaneous drainage, Endoscopic ultrasound (EUS)‐guided drainage, Endoscopic retrograde cholangiopancreatography (ERCP) with main pancreatic duct stenting if ductal disruption is demonstrated and pleuro‐mediastinal decortication in cases of organized collections or pleural complications [31]. Given the complex anatomical involvement and potential for serious complications, management of mediastinal pancreatic pseudocysts should always be individualized on a case‐by‐case basis, utilizing local expertise and a stepwise approach. In our patient, conservative management was initiated, including broad‐spectrum antibiotics to prevent secondary infections and octreotide, a somatostatin analogue, administered to reduce pancreatic secretions and promote pseudocyst resolution. Additionally, ultrasound‐guided pigtail catheter drainage of the right‐sided pleural effusion was performed to alleviate respiratory distress and improve oxygenation, resulting in significant clinical improvement. Although resource constraints and LMIC settings may limit access to advanced endoscopic or surgical interventions, appropriately selected conservative management combined with image‐guided drainage can still provide effective disease control and favorable clinical outcomes when applied within a structured, stepwise treatment framework as in the reported case.

## 5 Conclusion

Pancreatic pseudocysts are recognized complications of both acute and chronic pancreatitis, with mediastinal extension representing a rare but serious manifestation that can mimic other thoracic conditions. This case highlights the importance of considering pancreatic pseudocysts in the differential diagnosis of unexplained massive pleural effusion, particularly in patients with a history of alcohol use or pancreatitis symptoms. Accurate diagnosis relies heavily on imaging modalities such as contrast‐enhanced CT and MRCP, combined with clinical and biochemical correlation. Management should be tailored individually, ranging from conservative medical therapy to invasive drainage depending on symptom severity and anatomical complexity. In resource‐limited and low‐ and middle‐income settings, where access to advanced endoscopic or surgical interventions may be restricted, a pragmatic, stepwise approach emphasizing early diagnosis, supportive medical care, and image‐guided drainage can still yield favorable clinical outcomes. Early recognition and multidisciplinary coordination remain critical to preventing life‐threatening complications and optimizing patient recovery, as demonstrated in this case.

## Author Contributions

**Sachchu Thapa:** methodology, project administration, resources, visualization, writing – review and editing. **Bijay Kunwar:** conceptualization, writing – original draft, writing – review and editing. **Anup Ghimire:** conceptualization, investigation, methodology, validation, visualization, writing – review and editing. **Manthan Sanjay Shinde:** project administration, resources, writing – review and editing. **Ashok Kushwaha:** investigation, writing – review and editing. **Arjun Gaire:** validation, writing – review and editing. **Jagdish Kunwar:** supervision, writing – review and editing. **Bikash Bikram Adhikari:** project administration, supervision, writing – review and editing.

## Funding

The authors have nothing to report.

## Ethics Statement

Case reports are exempt from ethical approval in our institution, Bir Hospital, Kathmandu.

## Consent

Written informed consent was obtained from the patient for participation and publication of this case report and accompanying images. A copy of the written consent is available for review by the Editor‐in‐Chief of this journal on request.

## Conflicts of Interest

The authors declare no conflicts of interest.

## Supporting information


**Data S1:** ccr371745‐sup‐0001‐Supinfo1@Supplementary file S1.docx.

## Data Availability

All data generated or analyzed during this study are included in this published article.
