# Supplementary material for: Pancreatic Pseudocyst With Thoracic Extension: A Clinicoradiological Case Report
Source: Clin Case Rep. 2025 Dec 29;14(1):e71745. doi: 10.1002/ccr3.71745 (PMC12745899; doi:10.1002/ccr3.71745)
Supplement: Supplementary file 1 — Data S1: ccr371745‐sup‐0001‐Supinfo1@Supplementary file S1.docx. [file CCR3-14-e71745-s001.docx]

**A Timeline showing of events during the management**

| Test | Results | S. I. Units | Reference Range in SI Unit |
| --- | --- | --- | --- |
| ***Hematology Report*** | | | |
| CBC | | | |
| Total Count | | | |
| WBC | 7.5 | ×10⁹/L | 4.0–11.0 |
| RBC | 4.46 | ×10¹²/L | 4.7–6.0 |
| Platelets | 375 | ×10⁹/L | 150–400 |
| Differential Count | | | |
| Neutrophils | 43 | % | 40–70 |
| Lymphocytes | 44 | % | 20–45 |
| Monocytes | 10 | % | 2–10 |
| Eosinophils | 3 | % | 1–6 |
| Basophils | 0 | % | 0–1 |
| Hemoglobin | 139 | g/L | 130–180 |
| PCV | 0.42 | L/L | 0.40–0.54 |
| MCHC | 330 | g/L | 280–350 |
| MCH | 28 | pg | 27–32 |
| MCV | 81 | fL | 80–96 |
| ***Biochemistry Report*** | | | |
| Blood Urea | 2.66 | mmol/L | 1.67–7.50 |
| Serum Creatinine | 53 | µmol/L | 35–124 |
| Sodium | 129 | mmol/L | 135–145 |
| Potassium | 3.3 | mmol/L | 3.5–5.0 |
|  | | | |
| Serum Bilirubin Total | 13.7 | µmol/L | 5–17 |
| Serum Bilirubin Direct | 8.6 | µmol/L | 0–6.8 |
| SGPT/ALT | 30 | U/L | 5–40 |
| Alk. Phosphatase | 83 | U/L | 35–150 |
| SGOT/AST | 47 | U/L | <50 |
| Serum Total Protein | 61 | g/L | 45–80 |
| Serum Albumin | 29 | g/L | 25–55 |
| Lactate dehydrogenase | 208 | U/L | 225–450 |
| Serum Amylase | 492* | U/L | <80 |
| Serum Lipase | 198* | U/L | 0–38 |
| ***Microbiology Report*** | | | |
| Sputum for Gram stain | Gram positive and negative cocci, bacilli and others not seen | | |
| Sputum for AFB A/B/ GeneXpert | Not detected | | |
| ***Pleural fluid Analysis*** | | | |
| Culture sensitivity | No growth for organisms after 48 hours, inoculation at 37 degree C | | |
| Pleural Fluid Serum Amylase | 11,545* (fluid) | IU/L | <80 |
| Pleural Fluid repeated analysis | 15,535* (fluid) | IU/L | <80 |
| Pleural Fluid Protein | 45 | g/L | >30 g/L |
| Pleural/Serum Protein Ratio | 0.73* | Ratio | >0.5 |
| Pleural Fluid LDH | 420 | U/L |  |
| Pleural/Serum LDH Ratio | 2.02* | Ratio |  |
| Pleural Fluid Glucose | 2.8 | mmol/L | <3.3 mmol/L |
| Pleural Fluid pH | 7.25 | pH units | <7.3 |
| Pleural Fluid Total Cell Count | 3200* | cells/µL | >1000 |

**Table (1): Lab Reports On the day of admission**

*=abnormal value

**Table (2): Lab Reports after 3^rd^ and 5^th^ of admission**

| Test | Results | SI Unit | Reference Range in SI unit |
| --- | --- | --- | --- |
| ***Hematology Report*** | | | |
| CBC | | | |
| Total Count | | | |
| WBC | 19.9* | ×10⁹/L | 4.0–11.0 |
| RBC | 4.46 | ×10¹²/L | 4.7–6.0 |
| Platelets | 355 | ×10⁹/L | 150–400 |
| Differential Count | | | |
| Neutrophils | 82 | % | 40–70 |
| Lymphocytes | 16 | % | 20–45 |
| Monocytes | 2 | % | 2–10 |
| Eosinophils | 0 | % | 1–6 |
| Basophils | 0 | % | 0–1 |
| Hemoglobin | 141 | g/L | 130–180 |
| PCV | 0.43 | L/L | 0.40–0.54 |
| MCHC | 330 | g/L | 280–350 |
| MCH | 26 | pg | 27–32 |
| MCV | 78 | fL | 80–96 |
| ***Biochemistry Report*** | | | |
| Blood Urea | 5.33 | mmol/L | 1.67–7.50 |
| Serum Creatinine | 79.6 | µmol/L | 35–124 |
| Sodium | 131 | mmol/L | 135–145 |
| Potassium | 4.21 | mmol/L | 3.5–5.0 |
| Serum Bilirubin Total | 59.8 | µmol/L | 5–17 |
| Serum Bilirubin Direct | 30.8 | µmol/L | 0–6.8 |
| SGPT/ALT | 58 | U/L | 5–40 |
| Alkaline Phosphatase | 96 | U/L | 35–150 |
| SGOT/AST | 24 | U/L | <50 |
| Serum Total Protein | 55 | g/L | 45–80 |
| Serum Albumin | 26 | g/L | 25–55 |
| Lactate dehydrogenase | 208 | U/L | 225–450 |
| Sodium and Potassium levels on Day 5 of admission | | | |
| Sodium | 138.5 | mmol/L | 135–145 |
| Potassium | 4.21 | mmol/L | 3.5–5.0 |

| Test | Results | SI Units | Reference Range in SI unit |
| --- | --- | --- | --- |
| ***Hematology Report*** | | | |
| CBC | | | |
| Total Count | | | |
| WBC | 10.6 | ×10⁹/L | 4.0–11.0 |
| RBC | 4.32 | ×10¹²/L | 4.7–6.0 |
| Platelets | 527 | ×10⁹/L | 150–400 |
| Differential Count | | | |
| Neutrophils | 70 | % | 40–70 |
| Lymphocytes | 29 | % | 20–45 |
| Monocytes | 0 | % | 2–10 |
| Eosinophils | 1 | % | 1–6 |
| Basophils | 0 | % | 0–1 |
| Hemoglobin | 116 | g/L | 130–180 |
| PCV | 0.34 | L/L | 0.40–0.54 |
| MCHC | 340 | g/L | 280–350 |
| MCH | 27 | pg | 27–32 |
| MCV | 79 | fL | 80–96 |
| ***Biochemistry Report*** | | | |
| Blood Urea | 2.99 | mmol/L | 1.67–7.50 |
| Serum Creatinine | 61.9 | µmol/L | 35–124 |
| Sodium | 139 | mmol/L | 135–145 |
| Potassium | 3.6 | mmol/L | 3.5–5.0 |
| Serum Bilirubin Total | 15.4 | µmol/L | 5–17 |
| Serum Bilirubin Direct | 8.6 | µmol/L | 0–6.8 |
| SGPT/ALT | 27 | U/L | 5–40 |
| Alkaline Phosphatase | 151 | U/L | 35–150 |
| SGOT/AST | 26 | U/L | <50 |
| Serum Total Protein | 69 | g/L | 45–80 |
| Serum Albumin | 20 | g/L | 25–55 |

**Table (3): Lab Reports on 7^th^ day of admission**
